# Enriched enrollment randomized double-blind placebo-controlled cross-over trial with phenytoin cream in painful chronic idiopathic axonal polyneuropathy (EPHENE): a study protocol

**DOI:** 10.1186/s13063-022-06806-8

**Published:** 2022-10-22

**Authors:** David J. Kopsky, Ruben P. A. van Eijk, Janna K. Warendorf, Jan M. Keppel Hesselink, Nicolette C. Notermans, Alexander F. J. E. Vrancken

**Affiliations:** 1 Institute for Neuropathic Pain, Amsterdam / Soest / Bosch en Duin, The Netherlands; 2 Department of Neurology, Brain Centre University Medical Center Utrecht, Utrecht University, Utrecht, The Netherlands; 3 Biostatistics & Research Support, Julius Center for Health Sciences and Primary Care, University Medical Center Utrecht, Utrecht, The Netherlands

**Keywords:** Polyneuropathy, Neuropathic pain, Phenytoin, Cream, Topical, Randomized study, Cross-over trial, CIAP, Cryptogenic Polyneuropathy, Idiopathic polyneuropathy

## Abstract

**Background:**

Patients with chronic idiopathic axonal polyneuropathy (CIAP) can have neuropathic pain that significantly impacts quality of life. Oral neuropathic pain medication often has insufficient pain relief and side effects. Topical phenytoin cream could circumvent these limitations.

The primary objectives of this trial are to evaluate (1) efficacy in pain reduction and (2) safety of phenytoin cream in patients with painful CIAP. The main secondary objective is to explore the usefulness of a double-blind placebo-controlled response test (DOBRET) to identify responders to sustained pain relief with phenytoin cream.

**Methods:**

This 6-week, enriched enrollment randomized double-blind, placebo-controlled triple cross-over trial compares phenytoin 20%, 10% and placebo cream in 48 participants with painful CIAP. Enriched enrollment is based on a positive DOBRET in 48 participants who experience within 30 minutes ≥2 points pain reduction on the 11-point numerical rating scale (NRS) in the phenytoin 10% cream applied area and ≥1 point difference in pain reduction on the NRS between phenytoin 10% and placebo cream applied area, in favour of the former. To explore whether DOBRET has predictive value for sustained pain relief, 24 DOBRET-negative participants will be included.

An open-label extension phase is offered with phenytoin 20% cream for up to one year, to study long-term safety.

The main inclusion criteria are a diagnosis of CIAP and symmetrical neuropathic pain with a mean weekly pain score of ≥4 and <10 on the NRS.

The primary outcome is the mean difference between phenytoin 20% versus placebo cream in 7-day average pain intensity, as measured by the NRS, over week 2 in DOBRET positive participants.

Key secondary outcomes include the mean difference in pain intensity between phenytoin 10% and phenytoin 20% cream, and between phenytoin 10% and placebo cream. Furthermore, differences between the 3 interventions will be evaluated on the Neuropathic Pain Symptom Inventory, EuroQol EQ5-5D-5L, and evaluation of adverse events.

**Discussion:**

This study will provide evidence on the efficacy and safety of phenytoin cream in patients with painful CIAP and will give insight into the usefulness of DOBRET as a way of personalized medicine to identify responders to sustained pain relief with phenytoin cream.

**Trial registration:**

ClinicalTrials.gov NCT04647877. Registered on 1 December 2020.

## Administrative information

Note: the numbers in curly brackets in this protocol refer to the SPIRIT checklist item numbers. The order of the items has been modified to group similar items (see http://www.equator-network.org/reporting-guidelines/spirit-2013-statement-defining-standard-protocol-items-for-clinical-trials/).

**Table:** 

Title {1}	Enriched randomized double-blind, placebo-controlled cross-over trial with phenytoin cream in painful chronic idiopathic axonal polyneuropathy [EPHENE]: a study protocol
Trial registration {2a and 2b}.	Clinicaltrials.gov NCT04647877. Registered on 1 December 2020
Protocol version {3}	4 September 2020, version 1.9
Funding {4}	Prinses Beatrix Spierfonds, Dr. C.J. Vaillant Fonds
Author details {5a}	David J. Kopsky^1, 2^ Ruben P.A. van Eijk^2,3^ Janna K. Warendorf^2^ Jan M. Keppel Hesselink^1^ Nicolette C. Notermans^2^ Alexander F.J.E. Vrancken^2^ ^1^Institute for Neuropathic Pain, Amsterdam / Soest / Bosch en Duin the Netherlands. ^2^Department of Neurology, Brain Centre University Medical Center Utrecht, Utrecht University, Utrecht, the Netherlands. ^3^Biostatistics & Research Support, Julius Center for Health Sciences and Primary Care, University Medical Center Utrecht, Utrecht, the Netherlands.
Name and contact information for the trial sponsor {5b}	D.J. Kopsky, University Medical Center Utrecht, d.j.kopsky@umcutrecht.nl
Role of sponsor {5c}	The investigators are the study sponsors and responsible for the study design; collection, management, analysis, and interpretation of data; writing of the study report and submission for publication. The role of the study funders is limited to financial support.

## Introduction

### Background and rationale {6a}

Chronic idiopathic axonal polyneuropathy (CIAP) is a slowly progressive distal symmetric sensory or sensorimotor polyneuropathy without a known cause [1]. Sensory or sensorimotor symptoms start in the toes and gradually extend to the feet or lower legs, and sometimes involve the hands. Almost all patients experience negative sensory symptoms such as numbness and tightness (as if something is wrapped tightly around the foot or leg giving an unpleasant tense feeling of compression). Between 27–60% of CIAP patients report neuropathic pain in the feet sometimes extending into the lower legs, which impacts quality of life, work, sleep, and can induce or worsen depression in patients with polyneuropathy [2–5]. Painful positive sensory symptoms include tingling, pins and needles, burning. Up till now, no randomized controlled trials have been conducted in patients suffering from (painful) CIAP [1]. However, current first-line pharmacological treatment recommendations for painful polyneuropathy in general are oral tricyclic antidepressants, serotonin-noradrenaline reuptake inhibitors, and gabapentinoids [6–9]. A majority of patients have insufficient analgesic effect from currently available oral pain medication, but do experience mild, moderate or unacceptable side effects that hamper compliance to treatment [8, 10]. The number needed to treat (NNT) for oral tricyclic antidepressants, serotonin-noradrenaline reuptake inhibitors, and gabapentinoids varies between 3.6 and 7.7 for a treatment response of ≥ 50% pain reduction [8]. The number needed to harm (NNH) for treatment by the maximal daily dose ranges between 2.6 and 10.4 [10]. Especially in elderly patients, pharmacological management can be challenging due to age-related body changes affecting drug pharmacokinetics, and due to polypharmacy causing more frequent drug-related side effects, with greater potential to harm [11].

Thus, new treatment strategies are needed to improve neuropathic pain management with less side effects. Topical analgesics are an interesting therapeutic option, because they might influence only the nerve endings in the epidermis without reaching the bloodstream, thus resulting in fewer or no systemic side effects [12, 13]. Topical analgesics used to treat neuropathic pain are lidocaine 5% patch and capsaicin 8% patch, notwithstanding very low to moderate quality of the evidence, and thus are proposed as second-line treatments [8, 9, 14, 15]. Topical use of analgesics seems especially feasible in localized neuropathic pain, which has been defined as neuropathic pain characterized by consistent and circumscribed area(s) of maximum pain [16].

The newly developed phenytoin cream could fulfil the need of a novel neuropathic pain treatment [17–21]. Phenytoin 5% and 10% cream demonstrated analgesic effect in patients with neuropathic pain in the absence of systemic side effects, and there are some indications that 10% cream is more effective compared to 5% cream [17–19]. In daily practice observational studies phenytoin 20% cream was effective and neither side effects nor detectable phenytoin plasma levels were reported [22, 23]. No randomized clinical trial has compared phenytoin cream versus placebo nor compared phenytoin 20% versus 10% cream.

A randomized clinical trial evaluating topical clonidine in diabetic neuropathic pain patients found a more profound pain reduction when patients experienced more burning sensation after application of topical capsaicin, suggesting that treatment success might be dependent on the preservation of signal transduction of sensory nerves from the skin to the brain [24]. An alternative elegant way to evaluate the integrity of sensory nerves is to determine the analgesic effect of the active cream within 30 min of application in a double-blind placebo-controlled response test (DOBRET) [21]. A DOBRET is especially feasible in symmetrical polyneuropathy and localized neuropathic pain such as in painful CIAP, because a proper comparison can be made after application of the active cream on for example one foot and placebo cream on the other foot. With the DOBRET, initial responders to the active cream can be identified. Also, local allergic reactions or transient aggravation of pain can be ruled out. Given the absence of trials evaluating analgesics in patients with CIAP, we will conduct a DOBRET enriched enrollment randomized double-blind placebo-controlled triple cross-over trial to evaluate the effectiveness and safety of phenytoin cream in painful CIAP. After the 6-week cross-over trial, participants are offered to use phenytoin 20% cream in the 1-year open-label period, to evaluate long-term pain reduction and safety.

### Objectives {7}

The primary objectives are to evaluate (1) efficacy in pain reduction and (2) safety of phenytoin cream in participants with painful CIAP. Safety is evaluated by asking for local and systemic side effects, and by measuring phenytoin plasma levels. Possible reported systemic side effects will be correlated with phenytoin plasma levels. The secondary main objective is to explore the usefulness of DOBRET as a way of personalized medicine to identify responders to sustained pain relief with phenytoin cream.

### Trial design {8}

This is a 6-week single-centre, enriched enrollment randomized double-blind, placebo-controlled triple cross-over trial to evaluate the efficacy and safety of phenytoin cream (20% and 10%) versus placebo in 48 participants with painful CIAP. The enriched enrollment is based on a positive double-blind placebo-controlled response test (DOBRET) in 48 participants who experience within 30 minutes at least 2 points pain reduction on the 11-point numerical rating scale (NRS) on the phenytoin 10% cream applied area and at least 1 point difference in pain reduction on the NRS between phenytoin 10% and placebo cream applied area, in favour of the former. To explore to which extent and which responder definition for the DOBRET has a predictive value for sustained pain relief, an additional 24 DOBRET negative participants will be included.

**Fig. 1:**
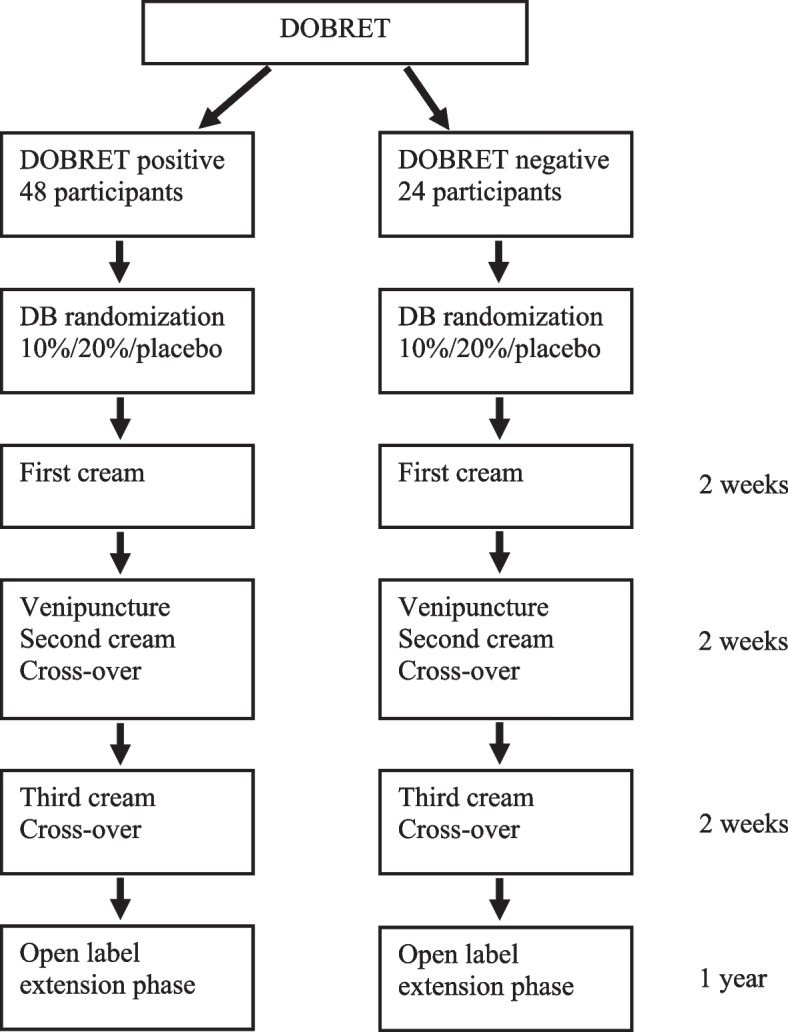
Flow chart. DB, double-blind; DOBRET, double-blind placebo-controlled response test

Participants will be randomly allocated to one of the 6 possible intervention sequences. Participants and investigators will be blinded. The duration of each treatment period is 2 weeks. Participants will cross-over two times to each of the other treatments. The study does not have wash-out periods between treatments, because the mean duration of the analgesic effect after an application is expected to be less than 9 h [19]. A flow chart of the study is presented in Fig. 1.

## Methods: participants, interventions and outcomes

### Study setting {9}

This monocentric trial will be executed in the University Medical Center Utrecht (UMCU), the Netherlands.

### Eligibility criteria {10}

Patients will be recruited from the UMCU neurology outpatient clinic. When patients from outside of the UMCU want to participate, a consultation for the confirmatory diagnosis will be carried out. Inclusion criteria are age ≥ 40 years; confirmed CIAP diagnosis [25]; presence of ≥3 months localized neuropathic pain in two symmetrical areas of feet/lower legs; with a pain intensity difference of ≤1 point on the NRS; ≥1 h daily pain; with a weekly mean pain score between ≥4 and <10 on the NRS at study entry; Douleur Neuropathique 4 questions (DN4) score ≥4 [26]; and no changes in neuropathic pain medication of ≥1 month. The main exclusion criteria are other (neuropathic) pain conditions; open wounds in the neuropathic pain area; current use of topical analgesics; hypersensitivity to study drugs; oral use of phenytoin and (planned) pregnancy. The primary investigator (AFJEV) and the two research physicians (JKW and DJK) will screen patients for eligibility. Eligible patients who are willing to participate will be recruited for the trial.

### Who will take informed consent? {26a}

Upon confirmation of willingness to participate, the eligible patient will be sent the patient information regarding the study and the informed consent form. One week later the research physician will phone the patient to answer any questions and will ask if the patient is willing to participate. Upon the patient’s agreement, the research physician will obtain the patients’ written informed consent at the first study visit.

### Additional consent provisions for collection and use of participant data and biological specimens {26b}

In the patient information letter, the trial outline is presented. During the trial, at the second visit blood withdrawal will be performed to determine phenytoin plasma concentrations in patients who applied 10% and 20% cream, in order to evaluate the safety of topical phenytoin. Possible reported systemic side effects will be correlated with phenytoin plasma levels. Plasma phenytoin analyses will be performed after data analyses to maintain blinding. The written consent form contains statements that the participant has read the information letter, could ask questions, had enough time of reflection on the trial, gives permission of collection and use of participant data, and can withdraw from the trial at any time.

## Interventions

### Explanation for the choice of comparators {6b}

In this cross-over, double-blind trial the comparator will be placebo cream applied 2 to 4 times daily according to the duration of effect. The active and placebo cream have the same white appearance and neutral smell. All participants will receive this treatment during a 2-week treatment period. Placebo cream is chosen to identify the magnitude of pain reduction attributable to the active interventions. Furthermore, a placebo cream is also used in the DOBRET to explore the predictive value of this test [21].

### Intervention description {11a}

All participants will receive placebo, phenytoin 10% and phenytoin 20% cream, each during a period of 2 weeks. The participant will be instructed to apply the cream 2 to 4 times a day with a maximum of 1.5 g per application, measured in Finger Tip Units (FTU).

**Fig. 2:**
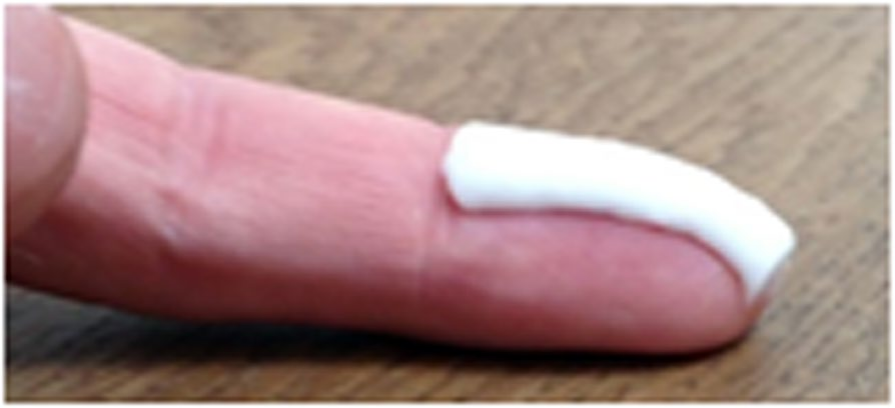
Finger Tip Unit

One FTU is defined as the amount of cream expressed from a tube with a 5 mm diameter nozzle, applied from the distal skin crease to the tip of the index finger, which amounts to approximately 0.5 g (see Fig. 2). The number of daily applications depends on the duration of the analgesic effect and the amount of cream depends on the size of the painful areas, e.g. both feet 1 FTU, and both feet and lower legs 3 FTU. The participant can apply the cream with bare hands, and then wash the hands after each application. After the double-blind period participants are invited to use phenytoin 20% cream in the 1-year open-label extension phase, to study the long-term pain reduction and safety.

### Criteria for discontinuing or modifying allocated interventions {11b}

Participants are allowed to drop out at any time without giving a reason. Other criteria are serious adverse event (SAE), unacceptable adverse event (AE), and/or protocol violation, including use of prohibited pain medication.

### Strategies to improve adherence to interventions {11c}

Participants can choose to fill in the pain diary and questionnaires electronically or on paper. Participants fill in every day the pain diary to score daily pain intensity on the NRS, number of daily cream applications and possibly used escape medication. Questionnaires are filled in only at the last day of each 14-day intervention period. When the pain diary is not filled in, the electronic questionnaire system Castor will send reminders the next day in the morning and the day after. Every 2 weeks the participant will visit the outpatient clinic to receive the study medication. The research physician checks at every visit the pain diary and questionnaires for completion.

In case the questionnaires are not filled in on the 14th day, the research physician will ask the participant to fill in the questionnaire at the visit on the 15th day.

When the pain diary is incomplete at the first visit, the research physician will stipulate the importance of filling in the pain diary and will monitor in Castor thereafter every day. In case the pain diary is not filled in, the patient will be phoned to ask the pain intensity on the NRS of the previous day, which will be noted, and will resolve the problem of poor adherence. When after the first 2 weeks less than 2 daily cream applications have been entered in the pain diary, the participant will be reinstructed during the second visit. The research physician will then monitor in Castor every day, and patients will be contacted in case of poor adherence. When poor adherence on paper diary and questionnaires is noticed in the first visit, daily phone calls will be performed in the second and third intervention periods. The outcomes will be registered in Castor and compared with the paper version. In case the participant experiences adverse events, one of the research physicians can be contacted for advice. Participants can contact the neurology department 24 h a day.

### Relevant concomitant care permitted or prohibited during the trial {11d}

Participants can continue their neuropathic pain medication when the dosage has been stable for at least one month. Acetaminophen and non-steroid anti-inflammatory drugs are allowed as escape medication for acute pain other than the neuropathic pain due to CIAP. Oral phenytoin, opiates and other topical analgesics are not allowed. Use of escape medication will be recorded in the on a daily base.

### Provisions for ancillary and post-trial care {30}

Participants can be reimbursed for transportation expenses. After the double-blind phase, participants can participate in a 1-year open-label extension phase. Hereafter, the general physician can prescribe phenytoin cream made by a compounding pharmacy. The UMCU has a liability insurance which is in accordance with article 7 of the Dutch law on medical research. This insurance provides cover for damage to research participants through injury or death caused by the trial. The insurance applies to the damage that becomes apparent during the trial or within 4 years after the end of the trial.

### Outcomes {12}

#### Primary outcome

The primary outcome is the mean difference between phenytoin 20% cream versus placebo cream in the 7-day average pain intensity, as measured by the NRS, over week 2 in DOBRET-positive participants.

#### Secondary outcomes

All secondary outcomes are evaluated for phenytoin 20% versus 10% versus placebo cream in DOBRET positive, negative and all participants.

The change between baseline and week 2 for each intervention is assessed for:

1. Pain intensity measured on the
  - NRS
  - Neuropathic Pain Symptom Inventory (NPSI), evaluating on the NRS neuropathic pain characteristics [27]
  - NRS for the 3 worst pain characteristics
  - ≥30% and ≥50% improvement on the NRS compared to placebo within one participant, reflecting at least moderate and at least substantial improvement [28]
  - Patient-reported percentage of pain reduction at the end of the second week of each intervention
  - Onset of the analgesic effect after application
  - Duration of the analgesic effect after one application
  - Daily number of cream applications
  - Use of escape pain medication
2. Quality of life measured on the
  - subscales of the Brief Pain Inventory (sBPI), evaluating on the NRS pain interference regarding general activity, mood, walking ability, normal work, relations with other people, sleep, and enjoyment of life [29]
  - EQ5-5D-5L, measuring the health status on the visual analogue scale, and on a 5-point scale pain/discomfort, anxiety/depression, mobility, self-care and usual activities [30]
3. Patient Global Impression of Change Scale (PGIC), measured on a 7-point scale [31]
4. To evaluate the safety and tolerability of phenytoin 10% and 20% cream
  - Tolerability is defined as time-to-discontinuation of an assigned treatment since randomization.
    Safety is based on the safety assessments including physical examinations, clinical laboratory evaluations, vital signs and frequency of AEs or SAEs. (S)AEs will be categorized according to the Common Terminology Criteria for Adverse Events and will be rated for severity and association with the study drug.
  - Detection of phenytoin in plasma
5. Predictive value of DOBRET in sustained pain reduction due to phenytoin cream
6. To evaluate the correlation between the PCS and the pain-reducing effect of topical phenytoin

### Participant timeline {13}

Participant timeline is shown in Table 1.

**Table 1:**
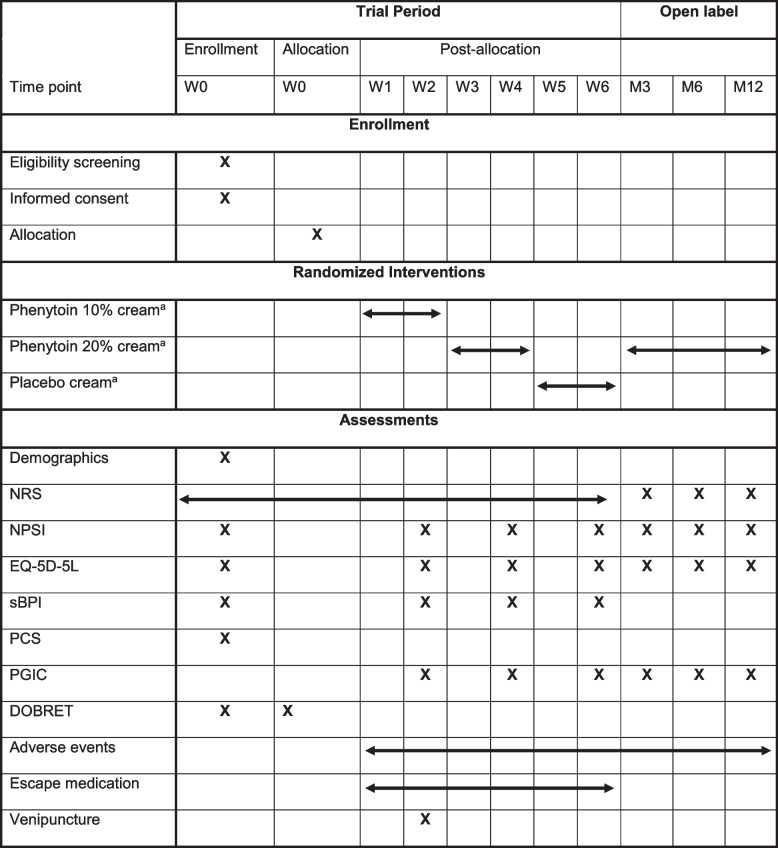
Time schedule of participant enrolment, allocation and assessments

*DOBRET* double-blind placebo-controlled response test, *EQ-5D-5L* euro quality of life questionnaire, *NPSI* neuropathic pain symptom inventory, *M* month, *NRS* 11-point numerical rating scale, *PCS* pain catastrophizing scale, *PGIC* patient global impression of change scale, *sBPI* subscales of brief pain inventory, *W* week

^a^The order of treatment will be randomized double-blindly

### Sample size {14}

We hypothesized a mean difference of 2 points on the NRS between placebo and the 20% cream (π1), and a mean difference of 1 point between 20% and 10% cream (π2). Based on preliminary data [21], we assumed for both treatment effects a standard deviation of 3.3 (standardized effect sizes: π1 = 0.61, π2 = 0.30). Using the same dataset, we conservatively assumed a correlation between NRS scores within participants (intraclass correlation) of 0.3. Seven participants per sequence (e.g. placebo–20%–10%, or 10%–placebo–20%) would be required to achieve 80% power and a two-sided significance level of 5% (= 7 × 6 sequences = 42 total sample size) [32]. Assuming a 10% drop-out rate, we will enrol 8 responding participants per group or 48 in total. DOBRET-negative participants are assumed to exhibit a mean difference of 1.5 point s (SD 4) on the NRS between placebo and the 20% cream, where 1 in 3 patients are assumed to be DOBRET-negative. This would result in a pooled overall treatment effect across DOBRET negative and positive participants of 1.83 (SD 4). Enrolling 24 DOBRET-negative and 48 DOBRET-positive participants would result in a power of 84% for the overall population.

### Recruitment {15}

On behalf of treating physicians, a standardized letter will be sent to patients who visited the neurology outpatient clinic of the UMCU from 2016 onwards and who were diagnosed with CIAP. When patients are interested to participate, they can contact the research physician, who will recruit the patients. The patients will be fully informed about the study, verbally and in writing, with special emphasis pertaining to the explanation of blood withdrawal for study purposes. The patients will be given at least one week to decide about their participation. Also, letters are sent to neurology departments of surrounding hospitals asking colleagues to refer eligible patients. On several websites such as the Princess Beatrix Muscle Foundation, the trial is described and interested patients can contact the outpatient clinic.

## Assignment of interventions: allocation

### Sequence generation {16a}

The pharmacist who prepares the study medication externally, according to Good Manufacturing Practice (GMP pharmacist) will randomly assign a label A, B or C to the tubes each containing 90 g of phenytoin 10%, 20% or placebo cream. For the DOBRET the GMP pharmacist will randomly assign a label A or B to the test tubes with phenytoin 10% cream and placebo cream. The final labelling on each study medication set of tubes will thus be S1A, S1B and S1C (first box), S2A, S2B and S2C (second box), and so on. The final labelling on each DOBRET tube set will thus be D1A and D1B (first box), D2A and D2B (second box), and so on. The study medication and DOBRET randomization list will be given by the GMP pharmacist to the UMCU pharmacist and only accessible by the UMCU pharmacist.

### Concealment mechanism {16b}

A blinded research physician will perform DOBRET, were after the results will be sent to the UMCU pharmacist, who will stratify participants according to the result of the DOBRET: 48 participants into the DOBRET positive arm, and 24 participants into the DOBRET negative arm. The tubes and cream are identical in appearance, to ensure the blinding of the participants and the treating physicians.

### Implementation {16c}

The GMP pharmacist is not involved in any other aspect of the study and will randomize the study medication in blocks of 6 with a randomization list generated with the Excel software programme. This will result in a balanced distribution of the labels A, B and C to the tubes with phenytoin 10%, 20% and placebo cream. The research physician will enrol the patients after performing DOBRET. The UMCU pharmacist will evaluate the outcome of the DOBRET, will stratify to DOBRET positive and DOBRET negative participants, and will randomly assign the participant to a study medication sequence (total of 6 possibilities, i.e. ABC, ACB, BAC, BCA, CAB, CBA) using a predefined randomization list.

## Assignment of interventions: blinding

### Who will be blinded {17a}

Participants, investigators, site personnel, and the study statistician are masked to treatment allocation. All randomized participants who received at least one dose of the study drug will be included in the analysis.

### Procedure for unblinding if needed {17b}

Only the UMCU pharmacist can unblind the creams in case of emergency. Unblinding can be performed 24 h a day.

## Data collection and management

### Plans for assessment and collection of outcomes {18a}

Demographic data and baseline assessments will be collected through an electronic or paper questionnaire. Medical history and past and current analgesic use with its effects will be asked at baseline visit or extracted from the UMCU electronic medical records. At baseline, at the 14th day of each treatment period, 3, 6 and 12 months of the open-label extension self-administered questionnaires will be collected (see for types of questionnaires Table 1). In the 6-week double-blind period, the NRS and the use of escape medication will be queried every evening. Adverse events will be asked and registered at each visit after a treatment period, and in the questionnaires in the open-label extension at 3, 6 and 12 months. The electronic questionnaires are automatically locked after completion. Research physician will enter the paper questionnaires into the electronic system and will store the paper questionnaires for 15 years. After completion of the trial and filling in all paper questionnaires, the electronic database will be locked. The analysis of phenytoin plasma levels will be performed with state-of-the-art liquid chromatography–mass spectrometry.

### Plans to promote participant retention and complete follow-up {18b}

Participants will be seen at the 14th day at every treatment period in the double-blind phase. Electronic pain diaries are stimulated to use over paper pain diaries and can be monitored continuously, and reminders will be sent electronically or will be given by phone when necessary. At 3, 6, and 12 months in the open-label extension phase, questionnaires will be sent electronically or on paper by post with a return envelope.

### Data management {19}

Pain diaries, questionnaires and research physicians’ case report forms will be directly stored in Castor, on a European server. Castor is an electronic data capture system for medical research [33]. Paper diaries and questionnaires will be securely 15 years stored in UMCU and entered into Castor by the research physicians.

### Confidentiality {27}

All participant data are stored pseudonymously. The participant coding list will be stored on a secured server and only research physicians, principal investigator, UMCU pharmacist, and independent auditor will have access. Informed consent forms, paper pain diaries and questionnaires will be stored in a locked closet, which can only be accessed by the principal investigator and research physicians. Participant information will be kept confidential.

### Plans for collection, laboratory evaluation and storage of biological specimens for genetic or molecular analysis in this trial/future use {33}

Blood samples will be collected in 1 ethylenediaminetetraacetic acid (EDTA) tube to measure free phenytoin (10%) and bound phenytoin (90%) in plasma. The specimen label will contain the participant study number. Blood samples will be stored at the UMCU laboratory, until the study is completed. The batch of blood samples from patients who were randomized during the first treatment period in the groups phenytoin 10% and 20% cream will be analysed to determine phenytoin plasma concentration. Unblinding of laboratory staff for treatment allocation will be done by the UMCU pharmacist. All samples will be destroyed after testing.

## Statistical methods

### Statistical methods for primary and secondary outcomes {20a}

Descriptive statistics will be used for baseline characteristics/demographics (age, sex, highest degree of education, country of birth, profession, duration and localization of pain, current neuropathic pain therapy, and past analgesic use), use of study creams (daily amount of use, number of daily applications, onset, duration and percentage of analgesic effect).

The primary endpoint measured on the NRS, as well as all other secondary endpoints, will be analysed according to the intention-to-treat (ITT) principle, incorporating all participants randomized irrespective of receiving treatment and having at least one efficacy measurement. For the primary analysis, the treatment effect will be determined in participants with a DOBRET-positive response at screening, evaluating the mean difference in NRS at week 2 between placebo and phenytoin 20% cream. Linear mixed-effects models (LME) will be used to account for the intra-individual clustering of observations. In brief, we will use an LME with fixed effects for time (week 1 or 2), treatment period (1 or 2 or 3), treatment (20% or 10% or placebo), the interaction between time and treatment, and NRS score at screening. The random part will be modelled with a random intercept and slope for time per individual, and an unstructured covariance matrix. The random slope for time will be kept in the model only if statistically significant. The likelihood ratio test will be used to test the significance of treatment; 95% confidence intervals will be based on the profile likelihood. Sensitivity analyses will be performed to evaluate (1) treatment-period interaction to assess potential carry-over effects and (2) per-protocol analysis involving all participants who completed the study. As secondary analysis, we will evaluate the treatment effect in all randomized patients irrespective of DOBRET, and explore the interaction between DOBRET outcome and treatment using additional interaction terms. Treatment effects on the NPSI, EQ-5D-5L, sBPI and PGIC will be evaluated in the same manner as the primary outcome.

Furthermore, the number of participants with a minimal pain relief (MPR), defined as the minimal percentage of pain reduction in the 7-day average pain intensity, as measured by the NRS, over week 2, will be calculated, for phenytoin 10%, phenytoin 20% and placebo cream. The number of participants experiencing the following MPRs will be determined:

- MPR30, at least 30% improvement as moderate pain improvement
- MPR50, at least 50% improvement as substantial pain improvement

Regression analyses will be performed to explore associations between DOBRET results and pain reduction during the intervention periods.

The statistical analysis will be performed with SPSS 22 (SPSS Inc., Chicago, IL, USA) and with the R-project, www.R-project.org (R Development Core Team (2008). R: A language and environment for statistical computing. R Foundation for Statistical Computing, Vienna, Austria. ISBN 3-900051-07-0).

### Interim analyses {21b}

No interim analyses will be performed.

### Methods for additional analyses (e.g. subgroup analyses) {20b}

As an exploratory objective, we will determine the test accuracy for DOBRET to identify participants who benefited from treatment, where treatment benefit is defined as a 30% reduction from baseline NRS. The following factors will be tested in the models for confounding effects: pain duration, and intensity, use of co-medication and escape medication, pain characteristics, pain catastrophizing score, level of education and duration of effect of study medication. Linear regression analyses will be used to evaluate the correlation of pain reduction with pain duration, and intensity, use of co-medication and escape medication, pain characteristics, catastrophizing, level of education and duration of effect. For all analyses, results will be considered significant when *p* < 0.05. For the subgroup analysis, we will correct the *p*-value according to Bonferroni.

### Methods in analysis to handle protocol non-adherence and any statistical methods to handle missing data {20c}

As LME models are flexible in handling missing outcome data when this occurs at random, we will not impute any missing efficacy endpoints for the primary analysis. As a sensitivity analysis, we will evaluate the following strategies: (1) last-observation-carried-forward and (2) jump-to-reference. Missing data in any of the covariates will be handled by multiple imputation and pooled according to Rubins’ rules.

### Plans to give access to the full protocol, participant level-data and statistical code {31c}

Participant information will be kept confidential. Results of the clinical trial will be published in a scientific journal, on the ClinicalTrials.gov website and presented on conferences. Data concerning the daily NRS will be publicly available. Requested additional data and/or analyses will be considered by the corresponding author of the published article.

## Oversight and monitoring

### Composition of the coordinating centre and trial steering committee {5d}

This monocentric trial has the following trial steering committee: principal investigator and two research physicians, supervising the trial process, such as recruiting participants and performing the trial, and reporting any AE and SAE to the Institutional Review Board. At trial completion, the trial steering committee and data manager decide in which journals the results of the trial will be published.

### Composition of the data monitoring committee, its role and reporting structure {21a}

Data monitoring will be carried out by an independent auditor, who will inspect the presence of the informed consent, verification of the inclusion and exclusion criteria, the completion of all data, the accountability of the study drug, and laboratory data. The auditor will brief any issues to the principal and coordinating investigators, who will address and resolve these issues and report back to the auditor.

### Adverse event reporting and harms {22}

All AEs and SAEs experienced by the trial participants will be monitored closely, properly documented and reported to the Institutional Review Board, which can terminate the trial immediately in case of serious life-threatening adverse events due to the study drug.

### Frequency and plans for auditing trial conduct {23}

The independent auditor will check the data after the first 5 inclusions, after the first participant completion of the 6-week double-blind period, and at closure of the trial. The auditor will inspect the process of data gathering.

### Plans for communicating important protocol amendments to relevant parties (e.g. trial participants, ethical committees) {25}

No changes in the execution of the trial according to the protocol are expected. In case of deviations in the execution of the protocol occur, detailed registration and reporting to the Institutional Review Board will be carried out. Any amendments to the protocol will be submitted to the Institutional Review Board for approval. After any approved amendment participants and all trial personnel will be informed.

### Dissemination plans {31a}

After completion trial results will be made available through scientific journals, clinicaltrials.gov and trialregister.nl websites.

## Discussion

Painful CIAP results from damage of the peripheral nerves, which become hyperexcitable as a consequence of upregulation and sensitization of receptors and ion channels [34]. For example, nerve damage results in the upregulation of voltage-gated sodium channels at the nerve endings and keratinocytes [35]. Also, the presence of proinflammatory molecules due to neuronal damage is part of the hyperexcitability of neurons. Peripheral sensitization due to damage of peripheral nerves can furthermore induce central sensitization [36].

Phenytoin can influence the upregulated and sensitized receptors and ion channels in peripheral neuropathic pain. For example, phenytoin inhibits voltage-activated sodium channels, leading to reduced firing of depolarized neurons [37]. Interestingly, phenytoin blocks sodium channels poorly at slow firing rates, allowing for normal activity, but suppresses the high-frequency repetitive firing leading to pain [37]. Voltage-dependent L-type calcium channels are also inhibited by phenytoin [38]. Furthermore, phenytoin potentiates GABA-induced current through modulation of the GABAA receptor in the nanomolar range [39]. Phenytoin (IC50 = 40 μM) has 6 times stronger sodium channel binding activity, compared to lidocaine (IC50 = 240 μM) [40]. Furthermore, inhibition of peripheral sensitization can diminish and/or abolish the signs and symptoms of central sensitization due to peripheral neuropathy [41]. Thus, topical phenytoin could also influence central sensitization. The hyperexcitable nociceptors reach in the epidermis up to the stratum corneum. Molecules smaller than 500 Dalton, such as phenytoin (252 Dalton) can easily penetrate this first barrier [42]. Therefore, topical phenytoin can have an analgesic effect within some minutes, and thus, a test application can be performed to reveal responders.

This trial will mainly be executed during the COVID-19 pandemic, which could affect recruitment due to patients’ fear to visit the out-patient clinic and due to capacity reduction of the out-patient clinic. This will partly be resolved by visiting patients at home. Neurological departments in the Netherlands will be approached for referral of patients. Furthermore, on the websites of Muscle disease (spierziekten.nl), Prinses Beatrix Spierfonds (spierfonds.nl), the expertise centre for CIAP (ciapexpertisecentrum.nl) and the Institute for Neuropathic Pain (neuropathie.nu) the study with its main inclusion criteria is described.

## Trial status

This protocol version 1.9, 4th of September 2020 has been approved by the Institutional Review Board Utrecht. The recruitment started on the 16th of December 2020 and is planned to be completed at the end of 2022.

## Data Availability

After completion of the trial, results will be presented in scientific journals and on the following websites:clinicaltrials.gov and trialregister.nl

## Abbreviations

AE: Adverse events
CIAP: Chronic idiopathic axonal polyneuropathy
DN4: Douleur Neuropathique 4 questions
DOBRET: Double-blind placebo-controlled response test
EDTA: Ethylenediaminetetraacetic acid
EQ-5D-5L: Euro quality of life questionnaire
FTU: Finger Tip Units
GMP: Good manufacturing practice
ITT: Intention to treat
LME: Linear mixed-effects models
MPR: Minimal pain relief
NPSI: Neuropathic pain symptom inventory
NRS: 11-point numerical rating scale
PGIC: Patient Global Impression of Change Scale
SAE: Serious adverse events
sBPI: Subscale of Brief Pain Inventory
UMCU: University Medical Center Utrecht
